# Polypharmacological target-aware molecule generation

**DOI:** 10.1093/bioinformatics/btag766

**Published:** 2026-10-12

**Authors:** Kehan Wu, Wei Zhang, Han Guo, Renhe Liu, Yu Shi, Peiran Jin, Haiguang Liu, Enhong Chen, Tao Qin, Zequn Liu, Yingce Xia

**Affiliations:** School of Computer Science and Technology, University of Science and Technology of China, Hefei, 230027, China; Zhongguancun Academy, Beijing, 100094, China; School of Computer Science and Technology, University of Science and Technology of China, Hefei, 230027, China; Zhongguancun Academy, Beijing, 100094, China; Global Health Drug Discovery Institute, Beijing, 100192, China; Global Health Drug Discovery Institute, Beijing, 100192, China; Zhongguancun Academy, Beijing, 100094, China; Zhongguancun Academy, Beijing, 100094, China; Zhongguancun Academy, Beijing, 100094, China; School of Computer Science and Technology, University of Science and Technology of China, Hefei, 230027, China; Zhongguancun Academy, Beijing, 100094, China; Zhongguancun Academy, Beijing, 100094, China; Zhongguancun Academy, Beijing, 100094, China

## Abstract

**Motivation:**

Designing compounds capable of modulating multiple target proteins simultaneously (i.e. polypharmacology) is critical for treating complex diseases such as cancer and neurodegenerative disorders. However, AI-driven multitarget drug design remains severely hindered by the scarcity of multitarget 3D structural data and the inefficiency of traditional “generate-then-filter” pipelines. There is a pressing need for scalable methods that can satisfy diverse binding constraints across multiple proteins without relying on scarce structural priors.

**Results:**

To overcome these bottlenecks, we reformulate multitarget drug design as a sequence-based generation task and introduce PolyTamGen, an end-to-end autoregressive framework. We first curate a large-scale ligand-centric bioactivity dataset from ChEMBL and BindingDB, comprising 1.6 million unique ligands and 4.9 million bioactivity entries. PolyTamGen encodes target protein sets using ESM3, compresses these high-dimensional features into compact tokens via an attention module, and feeds them into a sequence-based compound generator to produce the final multitarget ligands. We further apply Direct Preference Optimization to improve docking-based affinity proxies and drug-likeness. Extensive experiments on dual- and triple-target benchmarks demonstrate that PolyTamGen improves joint success rates over representative baselines while maintaining high chemical validity and diversity.

**Availability and implementation:**

The code is available at https://github.com/bjzgcai/PolyTamGen. The processed data, model checkpoints, and benchmark results are available at Zenodo: https://doi.org/10.5281/zenodo.20594128.

## 1 Introduction

Polypharmacological drug design aims to develop single molecules that simultaneously modulate multiple disease-relevant targets and is an important strategy in modern drug discovery (Nikolić *et al.* 2016, Tu *et al.* 2021). Compared with administering multiple agents in combination, a rationally designed multitarget ligand may simplify dosing, reduce pharmacokinetic complexity, and avoid some interaction risks associated with coadministered drugs. However, multitarget activity does not intrinsically improve safety and may introduce additional on-target or off-target liabilities. Thus, successful polypharmacological design requires precise control of the desired target profile, pharmacological balance, selectivity, and safety (Anighoro *et al.* 2014, Proschak *et al.* 2019). This strategy is particularly relevant to complex diseases such as cancer (Anighoro *et al.* 2014, Proschak *et al.* 2019) and neurodegenerative disorders such as Alzheimer’s disease, where biological networks can exhibit redundancy and robustness against single-node perturbations (Albertini *et al.* 2021, Hossain and Hussain 2025). It may also be useful in resistance-prone settings, including some kinase-targeted cancer therapies, where therapeutic escape can arise from compensatory pathway activation or mutation-mediated loss of sensitivity to single-target inhibition (Knight *et al.* 2010). However, the design of polypharmacological molecules presents a formidable geometric challenge: the molecule must simultaneously satisfy the distinct pharmacophoric and steric constraints of multiple binding pockets, effectively searching for a solution within the narrow, complex intersection of disparate chemical spaces (Chenthamarakshan *et al.* 2023).

Recent advances in generative models for structure-based drug design have shown remarkable promise in generating high-affinity binders for specific protein pockets (Guan *et al.* 2023, Peng *et al.* 2026, Wu *et al.* 2024). These models typically require pocket-specific structural data for training and inference. To extend them to multitarget settings, prior work has adopted a generate-then-filter sequential pipeline: candidates are generated for a primary target and subsequently filtered or reranked based on predicted affinity for secondary targets (Xue *et al.* 2024, Xia *et al.* 2025a). This approach can be inefficient because optimizing primarily for one target does not explicitly account for the structural constraints of the remaining targets, potentially leading to high rejection rates during downstream filtering. An alternative solution is to train end-to-end 3D generative models that jointly condition on multiple pockets. However, this approach faces a severe data bottleneck (Munson *et al.* 2024). While single-target data [e.g. PDBbind (Liu *et al.* 2015), CrossDocked (Francoeur *et al.* 2020)] is available, true multitarget structural data, where the same ligand is cocrystallized with multiple different proteins, is extremely scarce. In our PDB-derived curation, we identified 2047 such ligands (Table 6, available as supplementary data at *Bioinformatics* online). Moreover, the dedicated multitarget structural database MTLD contains a similarly small number of entries (Chen *et al.* 2017). Such data scarcity creates a major bottleneck for structure-based approaches.

Sequence-based design offers a robust alternative by operating effectively without explicit 3D priors. These methods typically generate ligand representations, such as SMILES strings, conditioned directly on protein amino acid sequences (Grechishnikova 2021, Qian *et al.* 2022). Bioactivity databases for sequence-based design, such as ChEMBL (Mendez *et al.* 2019) and BindingDB (Gilson *et al.* 2016), contain millions of assay records, covering a vast chemical space. Although current sequence-based models are primarily designed for single-target generation (Qian *et al.* 2022), the data itself exhibits broader applicability. In particular, many ligands are associated with assay results from multiple distinct targets, demonstrating the potential of leveraging such data for multitarget compound design.

To this end, we introduce PolyTamGen (*Poly*pharmacological *T*arget-*A*ware *M*olecule *Gen*eration), a framework for multitarget *de novo* ligand generation. We first curate a large-scale ligand-centric bioactivity dataset by integrating ChEMBL and BindingDB and aggregating records into ligand-centric profiles. This yields a dataset comprising 1.6 million unique ligands and 4.9 million bioactivity entries, including profiles with activity annotations across multiple targets. Building on this dataset, PolyTamGen models the joint generation of ligands conditioned on sets of protein sequences. We encode each target using the ESM3 protein language model (Hayes *et al.* 2025), compress the resulting representations through a lightweight adaptor (Li *et al.* 2023), and model target interdependencies to capture their interactions. These fused target representations are fed into NatureLM (Xia *et al.* 2025b), a sequence-based molecular generation model, to produce ligands optimized for multiple targets. We further refine PolyTamGen using Direct Preference Optimization (DPO; Rafailov *et al.* 2023) to improve docking-based affinity proxies and drug-likeness. To evaluate our method, we construct a benchmark designed to generate compounds that can bind to two targets (the T2 setting) and three targets (the T3 setting) simultaneously. Under this common evaluation protocol, PolyTamGen achieves stronger joint compatibility metrics than the current baselines.

Our contributions are summarized as follows:

1. *Sequence-based joint-conditioning framework*: we present a unified architecture that injects ESM3-encoded protein constraints as continuous tokens into NatureLM. By framing multitarget design as a cross-modal translation task, our model explicitly conditions on target sets and captures cross-target dependencies within a scalable sequence-based generator.
2. *Comprehensive bioactivity dataset*: we curate a large-scale ligand-centric bioactivity dataset by merging ChEMBL and BindingDB, comprising 4.9 million bioactivity entries and 1.6 million unique ligands. This provides broad supervision across single- and multitarget activity profiles while bypassing the bottleneck of scarce 3D cocrystal structures.
3. *Benchmark and empirical analysis*: we establish dual-target (T2) and triple-target (T3) benchmarks for joint multitarget molecule generation. At the primary −8.18 kcal/mol cutoff, PolyTamGen-DPO exceeds the strongest external baseline by 16.8 and 25.9 percentage points on T2 and T3, respectively. Additional analyses further support the gains in target balance and architectural contribution.

### 1.1 Related work

Molecular generation has increasingly adopted language-model formulations in which SMILES strings are generated as chemical sequences. Early recurrent models and recent transformer-based systems learn chemical syntax from large corpora, while conditional variants add target, property, or instruction information to steer generation toward useful compounds (Arús-Pous *et al.* 2019, Bagal *et al.* 2022, Wang *et al.* 2023, Ye 2024, Xia *et al.* 2025b). In parallel, protein-conditioned and structure-based generators such as Pocket2Mol, PocketXMol, DecompDiff, and TamGen use pocket geometry or protein representations to improve target specificity (Peng *et al.* 2022, 2026; Guan *et al.* 2023; Wu *et al.* 2024). Preference- or reinforcement-based methods further optimize generated molecules using docking, affinity, or energy-related feedback (Rafailov *et al.* 2023, Cheng *et al.* 2025, Gu *et al.* 2024, Huang and Zhang 2025, Zhang *et al.* 2025).

Multitarget molecule design is less explored. Existing work often relies on implicit target-pair fine-tuning or predictor-driven multiobjective optimization, including reinforcement learning, simulated annealing, and evolutionary search (Isigkeit *et al.* 2024, Munson *et al.* 2024, Xue *et al.* 2024, Xia *et al.* 2025a). These approaches are valuable but typically require separate predictor-generator loops, fixed target-pair settings, or *post hoc* filtering. PolyTamGen instead learns a ligand-centric sequence model that conditions directly on target sets at inference time, while retaining explicit diagnostics for target balance, selectivity stress, and computational-validation limits. A closely related direction is LLM-based 3D multitarget molecule generation. LaMGen (Su *et al.* 2026) represents ligands with rotation-aware 3D tokens and constructs a conformation-aware multitarget dataset to generate energetically plausible 3D molecular conformations conditioned on multiple protein sequences. In contrast, PolyTamGen focuses on a complementary sequence-based setting: it learns joint target constraints from large-scale ligand-centric bioactivity records and generates SMILES conditioned on variable target sets.

### 1.2 Data construction

We construct a large-scale, ligand-centric dataset derived from biological activity records to train our model on diverse multitarget constraints. Formally, each training sample is defined as a tuple $\left(y,T_{\text{act}}\right)$, where y denotes the ligand SMILES string and $T_{\text{act}}$ represents the set of target proteins for which the ligand is confirmed active.

We aggregate bioactivity records from ChEMBL (Mendez *et al.* 2019) and BindingDB (Gilson *et al.* 2016) into a unified schema indexed by ligand and target identifiers. To ensure label reliability and resolve experimental noise, we implement a rigorous filtering pipeline:

1. *Standardization*: we retain only entries with high-confidence bioactivity types (e.g. IC_50_, $K_{i}$, $K_{d}$, and EC_50_) or percentage-based inhibition assays. All concentration values are normalized to nanomolar (nM) units. Records with ambiguous units or inconclusive qualitative descriptions are discarded.
2. *Binarization for model preprocessing*: we derive binary labels (1 for Active, 0 for Inactive) from the retained assay records. A ligand is considered active if it satisfies either of the following criteria: (i) a binding affinity ≤10 µM (10 000 nM) or (ii) an inhibition rate ≥50% in functional assays. Conversely, entries with affinities >10 µM, inhibition rates <50%, or explicit “inactive” annotations are labeled as inactive. The released record-level table preserves the activity type and, where reported, the numerical activity value and unit alongside the derived binary label. Binarization is applied only to construct the training data for the current model, while the original activity measurements are retained in the released dataset.
3. *Conflict resolution*: we deduplicate records based on the triplet of (Ligand SMILES, Target ID, Activity Label). For ligand-target pairs with conflicting binary labels (e.g. labeled as “Active” in one assay but “Inactive” in another due to differing experimental conditions), we discard the pair to prevent label noise.

After filtering and conflict resolution, the remaining records are aggregated by ligand to form ligand-centric bioactivity profiles, denoted as $D_{\text{bio}}$. $D_{\text{bio}}$ contains 4.87 million curated ligand–target activity records covering 1.65 million unique ligands. For each ligand, experimentally supported active and inactive target annotations are stored separately, while ligand–target pairs without experimental measurements are removed. The multitarget profiles are constructed from the set of active targets associated with the same ligand.

We partition $D_{\text{bio}}$ at the ligand level using Bemis–Murcko scaffolds, assigning ligands with the same scaffold to the same partition. The training ($D_{\text{bio}}^{\text{tr}}$) and validation ($D_{\text{bio}}^{\text{val}}$) partitions are used in the supervised fine-tuning (SFT) stage. The structural T2 and T3 benchmarks are constructed separately from $D_{\text{PDB}}$ and are not derived from a held-out subset of $D_{\text{bio}}$.

Separately, we construct a structural dataset, $D_{\text{PDB}}$, from PDB cocrystal structures by collecting ligands observed across multiple target structures. $D_{\text{PDB}}$ contains 2047 ligand-centric profiles and 21 040 ligand–target structure records, representing 7389 mapped canonical UniProt accessions. This dataset provides the resolved protein structures needed for docking-based optimization and evaluation. Its training ($D_{\text{PDB}}^{\text{tr}}$) and validation ($D_{\text{PDB}}^{\text{val}}$) partitions are used during the DPO stage, while its test partition ($D_{\text{PDB}}^{\text{test}}$) is used to construct the structural benchmarks.

Specifically, we construct two evaluation settings from $D_{\text{PDB}}^{\text{test}}$: (i) dual-target setting (denoted as T2), in which each generated molecule is expected to bind simultaneously to two targets; and (ii) triple-target setting (denoted as T3), in which it is expected to bind to three targets. The dual-target benchmark contains 151 target-pair contexts, comprising 288 unique pockets, and 113 reference ligands, while the triple-target benchmark contains 59 target-triplet contexts, comprising 97 unique pockets and 23 reference ligands. These benchmarks are organized around target/pocket combinations rather than ligand scaffolds or individual protein identities. Although T3 is conceptually more constrained than T2, the two benchmarks are different benchmark populations, so their headline metrics should not be interpreted as a strict monotonic ordering of difficulty. Additional statistics and analyses are provided in the Supplementary Information, available as supplementary data at *Bioinformatics* online.

## 2 Materials and methods

### 2.1 Network architecture

*Problem definition*: let $T=\{T_{1},\ldots ,T_{K}\}$ denote a set of *K* target protein sequences, where the *i*th protein $T_{i}$ consists of a sequence of amino acids $T_{i}=(a_{1},\ldots ,a_{L_{i}})$. The objective is to generate a valid molecule y, represented as a SMILES string $y=(y_{1},y_{2},\ldots ,y_{\left|y\right|})$ with |y| tokens, that can simultaneously bind to all proteins in T.

PolyTamGen is an end-to-end generative framework designed to model the conditional distribution p(y|T). As illustrated in Fig. 1, the architecture comprises four distinct modules: (i) a protein encoder for residue-level feature extraction, (ii) a protein adaptor for feature compression, (iii) a set-wise interaction module for cross-target information fusion, and (iv) an autoregressive SMILES generator.

**Figure 1:**
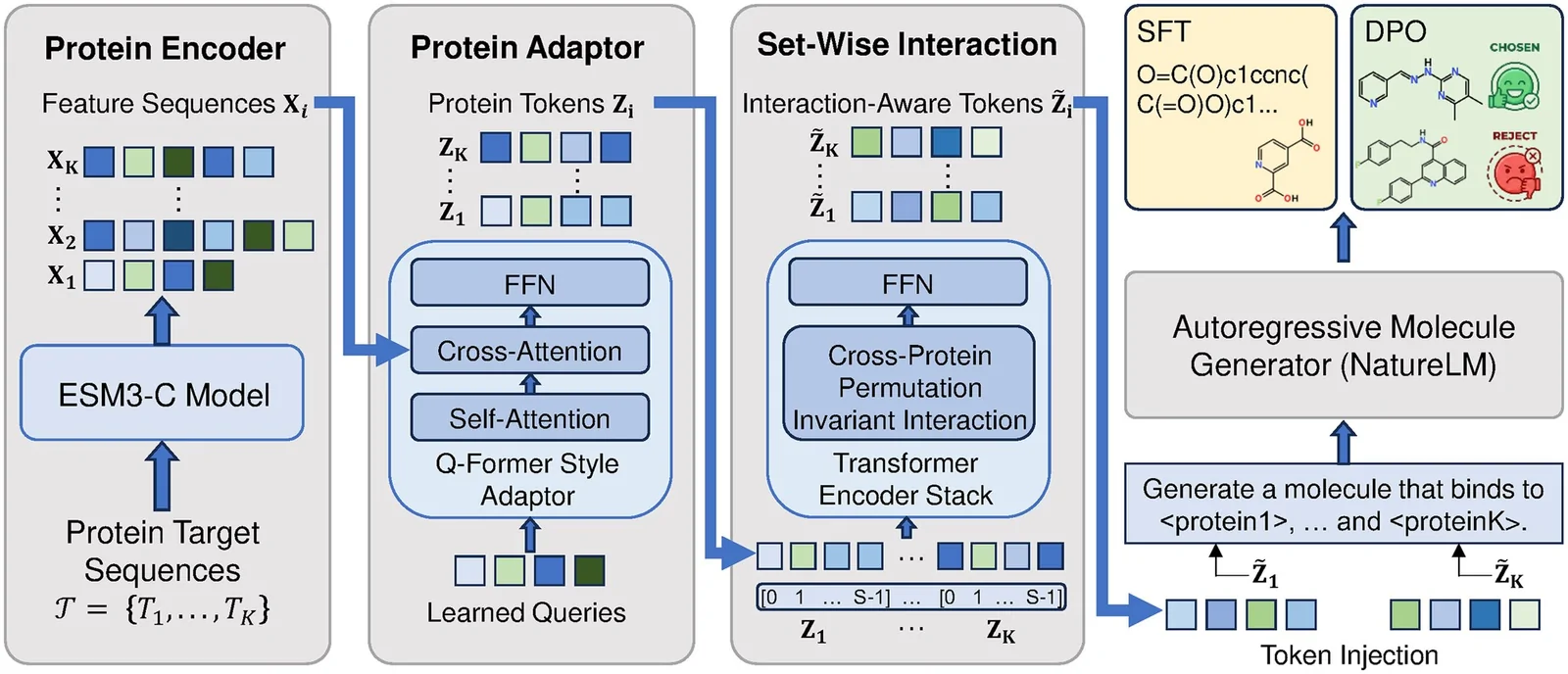
Overview of the PolyTamGen framework. The architecture consists of four main components: (i) Protein feature extraction: a frozen ESM3 backbone extracts feature sequences from the target proteins. (ii) Protein adaptor: a Q-Former-style adaptor employs a Transformer decoder to compress variable-length protein features into a fixed number of learned tokens, utilizing both self-attention among queries and cross-attention with protein features. (iii) Set-wise interaction: a Transformer encoder allows explicit information exchange between tokens of different proteins to satisfy multitarget constraints. (iv) Token injection and generation: the resulting interaction-aware tokens are injected into the natural language prompt, conditioning the autoregressive LLM [here we use NatureLM (Xia *et al.* 2025b)] to generate the target SMILES sequence. PolyTamGen is trained through SFT followed by DPO.

*Protein feature extraction*: for each target protein $T_{i}\in T$, we utilize the ESM3 protein language model (Hayes *et al.* 2025) as a frozen backbone to extract rich biological priors. This yields a residue-level feature matrix $X_{i}\in R^{L_{i}\times d_{\text{in}}}$, where $L_{i}$ is the sequence length and $d_{\text{in}}$ is the hidden dimension of ESM3.

*Protein adaptor*: given that protein sequences are typically long, often spanning hundreds or even thousands of residues, we employ an adaptor based on Q-Former (Li *et al.* 2023) to compress each protein’s feature sequence $X_{i}$ into a compact representation $Z_{i}$. Specifically, we initialize *S* learnable query embeddings $Q\in R^{S\times d_{\text{LLM}}}$. We then apply a transformer decoder architecture comprising self-attention, cross-attention, and feed-forward layers (Vaswani *et al.* 2017). The self-attention and feed-forward layers follow the standard transformer design; however, for the cross-attention layer, the keys and values are derived from the protein features $X_{i}$. By taking the output from the final layer of this adaptor, we obtain a compressed representation $Z_{i}\in R^{S\times d_{\text{LLM}}}$.

*Set-wise interaction*: the features in $Z_{i}$ are encoded independently for each protein. To capture dependencies among all target proteins, we introduce an explicit cross-target information flow inspired by the Set Transformer (Lee *et al.* 2019). We concatenate the features ${\left\{Z_{i}\right\}}_{i=1}^{K}$ and process them via a self-attention block. Within this interaction module, Rotary Positional Embedding (RoPE) indices (Su *et al.* 2024) are reset for each protein block so that different targets are not assigned distinct absolute block-position indices solely because of their order in the concatenation. Formally, this module transforms the input into interaction-aware tokens ${\left\{{\tilde{Z}}_{i}\right\}}_{i=1}^{K}$ of identical dimensions.

*Incorporating protein features into the molecule generator*: we employ NatureLM (Xia *et al.* 2025b) as the generative backbone. The continuous embeddings $\tilde{Z}$ are injected into the text prompt via soft-prompting. Specifically, we construct a prompt template “Generate a molecule that binds to {protein 1}, {protein 2}, …, {protein  *K*},” where placeholders are replaced by the corresponding active embeddings from $\tilde{Z}$. The model then generates the molecule *y* autoregressively:


(1) $$P_{\theta }(y|T)=\prod_{j=1}^{\left|y\right|}P_{\theta }(y_{j}|y_{<j},\tilde{Z}),$$

where θ denotes the parameters of our model. Module-level ablations in Table 4, available as supplementary data at *Bioinformatics* online, provide complementary evidence for the contributions of the set-wise interaction module and the Q-Former-style adaptor.

### 2.2 Training strategy

Our training framework proceeds in two stages: (i) SFT to learn the conditional distribution of bioactive molecules and (ii) DPO to align the model with docking-based affinity proxies and drug-likeness objectives.

*Supervised fine-tuning*: following the notations in Section 1.2, we denote the training set as $D_{\text{bio}}^{\text{tr}}={\left\{(y_{i},T_{\text{act},i})\right\}}_{i=1}^{N}$. To expose the model to variable target-set contexts, we employ a stochastic multitarget sampling strategy. Specifically, during each training iteration, parameters are optimized using a randomly sampled subset $T_{\text{act},i}^{\prime }\subseteq T_{\text{act},i}$ as the conditioning context for ligand $y_{i}$. Mathematically,


(2) $$L=-\frac{1}{\left|D_{\text{bio}}^{\text{tr}}\right|}\sum_{(y,T_{\text{act}})\in D_{\text{bio}}^{\text{tr}}}\frac{1}{\left|y\right|}\sum_{j=1}^{\left|y\right|}\;\text{log}\;P_{\theta }(y_{j}|y_{<j},T_{\text{act}}^{\prime }),$$

where $T_{\text{act}}^{\prime }$ is a randomly sampled subset of $T_{\text{act}}$, and $y_{<j}$ denotes $\left(y_{1},y_{2},\ldots ,y_{j-1}\right)$.

Crucially, this stochastic sampling functions as a form of combinatorial data augmentation. By dynamically varying the conditioning set, the training procedure encourages flexible constraint satisfaction rather than memorization of static target sets observed in the database. The explicit inclusion of single-target samples also helps preserve sensitivity to individual target constraints in multitarget contexts.

### 2.3 Direct preference optimization

We further apply DPO to improve docking-based affinity proxies and drug-likeness.

For any given molecule y and target set T, we define the reward function as follows:


(3) $$R(y,T)=-S_{\text{avg-dock}}(y,T)+\lambda \cdot \text{QED}(y),$$

where $S_{\text{avg-dock}}$ is the average Vina docking score across all targets in T (lower is better), and λ is a hyperparameter weighting the Quantitative Estimate of Drug-likeness (QED). For the reported DPO model, we use λ=5. Synthetic accessibility (SA) is not included in the reward.

Let $\pi_{\text{ref}}$ denote the model obtained from the SFT stage. For each instance $(y,T)\in D_{\text{PDB}}^{\text{tr}}$, we use $\pi_{\text{ref}}$ to generate a set of candidate molecules based on T. From these samples, the molecule achieving the highest reward is designated as the winner $y_{w}$, and the one with the lowest reward as the loser $y_{l}$, thereby forming a preference pair $\left(y_{w},y_{l}\right)$.

We then optimize the policy $\pi_{\theta }$ to assign higher probability to $y_{w}$ compared to $y_{l}$ while remaining close to the reference policy $\pi_{\text{ref}}$. The DPO loss is defined as follows:


(4) $$\begin{array}{cc} & L_{\text{DPO}}(\theta )=-E_{((y_{w},y_{l}),T)∼D_{\text{PDB}}^{\text{tr}}}\\ & \left[\text{log}\;\sigma \left(\beta \;\text{log}\;\frac{\pi_{\theta }(y_{w}|T)}{\pi_{\text{ref}}(y_{w}|T)}-\beta \;\text{log}\;\frac{\pi_{\theta }(y_{l}|T)}{\pi_{\text{ref}}(y_{l}|T)}\right)\right], \end{array}$$

where σ is the sigmoid function and β controls the strength of the preference log-ratio relative to the reference policy.

## 3 Results

### 3.1 Experimental setup

We first train PolyTamGen through SFT on $D_{\text{bio}}^{\text{tr}}$ and then refine it with DPO on $D_{\text{PDB}}^{\text{tr}}$. We evaluate the resulting models on the T2 and T3 benchmarks.

*Baselines*: to evaluate performance against existing paradigms, we benchmark PolyTamGen against two categories of baselines:

1. *External widely used baselines*: we select representative methods across different modalities:
  - TamGen (sequence-based; Wu *et al.* 2024): a Transformer model for target-specific generation, which has been verified through several real-world applications;
  - PocketXMol (structure-based; Peng *et al.* 2026): a representative 3D generative model conditioning on explicit pocket geometry.
    As these models are restricted to single-target generation, we adopt a “generate-then-pool” protocol for multitarget evaluation. For a target set containing multiple proteins, each single-target model generates candidates independently for each protein. We then combine the candidates generated for all individual targets into a single candidate set and evaluate every candidate against all intended targets. This evaluate-all setting is used as our primary comparison.
2. *Internal baselines*: we design two types of internal baselines to isolate the contributions of our joint training strategy and the DPO refinement stage.
  - PolyTamGen-Single: to isolate the efficacy of our joint training strategy, we train the same architecture (NatureLM + ESM3 Adaptor) exclusively on single-target bioactivity data. Inference follows the “generate-then-pool” protocol, serving as a controlled baseline for the multitarget objective;
  - PolyTamGen-SFT: this represents the implementation of our method trained solely via SFT, excluding the DPO stage.

*Evaluation Metrics*: we evaluate the generated molecules from the following perspectives:

1. *AvgDock (* ↓ *)*: the average Vina score (kcal/mol) across all targets in T;
2. *Strict success rate (* ${\text{SR}}_{\text{strict}}$ *) (* ↑ *)*: following DecompDiff (Guan *et al.* 2023), ${\text{SR}}_{\text{strict}}$ is the fraction of molecules with finite AvgDock values that simultaneously satisfy QED>0.25, ${\text{SA}}_{\text{norm}}>0.59$ and AvgDock <−8.18 kcal/mol.
3. *Drug-likeness and feasibility metrics*: we report Validity, Diversity (IntDiv), QED, and normalized SA, with ${\text{SA}}_{\text{norm}}=1-({\text{SA}}_{\text{raw}}-1)/9$ so that higher values indicate greater predicted accessibility. SA is not an explicit term in the DPO reward.

### 3.2 Benchmark evaluation

Table 1 presents the evaluation results for the T2 benchmark. Under the present docking-based evaluation protocol, PolyTamGen achieves the lowest AvgDock and the highest Strict Success Rate among the compared methods while maintaining competitive validity and diversity. We emphasize that these comparisons should be read as benchmark results under a common protocol, not as definitive proof of experimentally validated polypharmacology or a universal sequence-versus-structure claim. We have the following observations:

**Table 1:** Overall results on the T2 benchmark (generating ligands that bind to two target proteins simultaneously).^a^

Method	AvgDock	SR_strict@-8.18	Validity	Diversity	QED	SA
TamGen	−6.536 ± 0.997	0.073 ± 0.110	0.998 ± 0.003	0.863 ± 0.008	0.495 ± 0.045	0.717 ± 0.013
PocketXMol	−6.581 ± 1.503	0.163 ± 0.203	**0.999** ± **0.004**	0.863 ± 0.047	0.533 ± 0.081	0.787 ± 0.063
PolyTamGen-Single	−6.989 ± 0.877	0.148 ± 0.157	0.936 ± 0.030	**0.878** ± **0.007**	0.407 ± 0.044	0.746 ± 0.021
PolyTamGen-SFT	−7.347 ± 1.017	0.226 ± 0.233	0.959 ± 0.046	0.864 ± 0.025	0.408 ± 0.084	0.753 ± 0.048
PolyTamGen-DPO	−**7.642** ± **1.064**	**0.331** ± **0.330**	0.968 ± 0.110	0.837 ± 0.041	**0.565** ± **0.069**	**0.805** ± **0.032**

a Entries report mean ± SD across target pairs. Bold values indicate the best mean performance for each metric: lower AvgDock and higher values for all other metrics.

1. *Inefficiency of the “generate-then-pool” paradigm.* Under the evaluate-all generate-then-pool protocol, the strict success rates at the primary −8.18 kcal/mol cutoff are 7.3% for TamGen, 16.3% for PocketXMol, and 14.8% for PolyTamGen-Single, compared with 33.1% for PolyTamGen-DPO.
  Because the single-target baselines generate candidates independently for each target, they may produce more molecules than PolyTamGen under the same per-target sampling budget. We therefore additionally perform budget-matched comparisons using Random-*N*, which randomly samples the same number of candidates across methods, and Top-*N*, which selects the *N* candidates with the best docking scores. PolyTamGen achieves the best performance under both settings (see Fig. 2 and Table 2, available as supplementary data at *Bioinformatics* online, for more details).
2. *Efficacy of DPO.* The integration of DPO (PolyTamGen-DPO) improves the joint binding-oriented metrics relative to PolyTamGen-SFT. Specifically, ${\text{SR}}_{\text{strict}}$ increases from 22.6% to 33.1%, while AvgDock improves from −7.347 to −7.642 kcal/mol. Interestingly, normalized SA also increases from PolyTamGen-SFT to the reported PolyTamGen-DPO model, although SA is not explicitly included in the DPO reward. We therefore treat this as an empirical association rather than a directly optimized effect; QED and normalized SA show moderate molecule-level correlations across the evaluated molecules (T2: Pearson r=0.428, Spearman ρ=0.414; T3: Pearson r=0.416, Spearman ρ=0.411).
3. *Affinity check by AI predictors.* In addition to docking, we use FlexRibbon (Zhu *et al.* 2026), an external AI-based compound–target affinity predictor, to provide an independent check of binding affinity. FlexRibbon predicts $-\text{log}({\text{IC}}_{50})$, where ${\text{IC}}_{50}$ is measured in μM; therefore, larger values indicate stronger predicted binding. We perform this affinity-prediction check on the T2 benchmark. For each generated molecule, we average the predicted affinities over the two intended targets.

**Figure 2:**
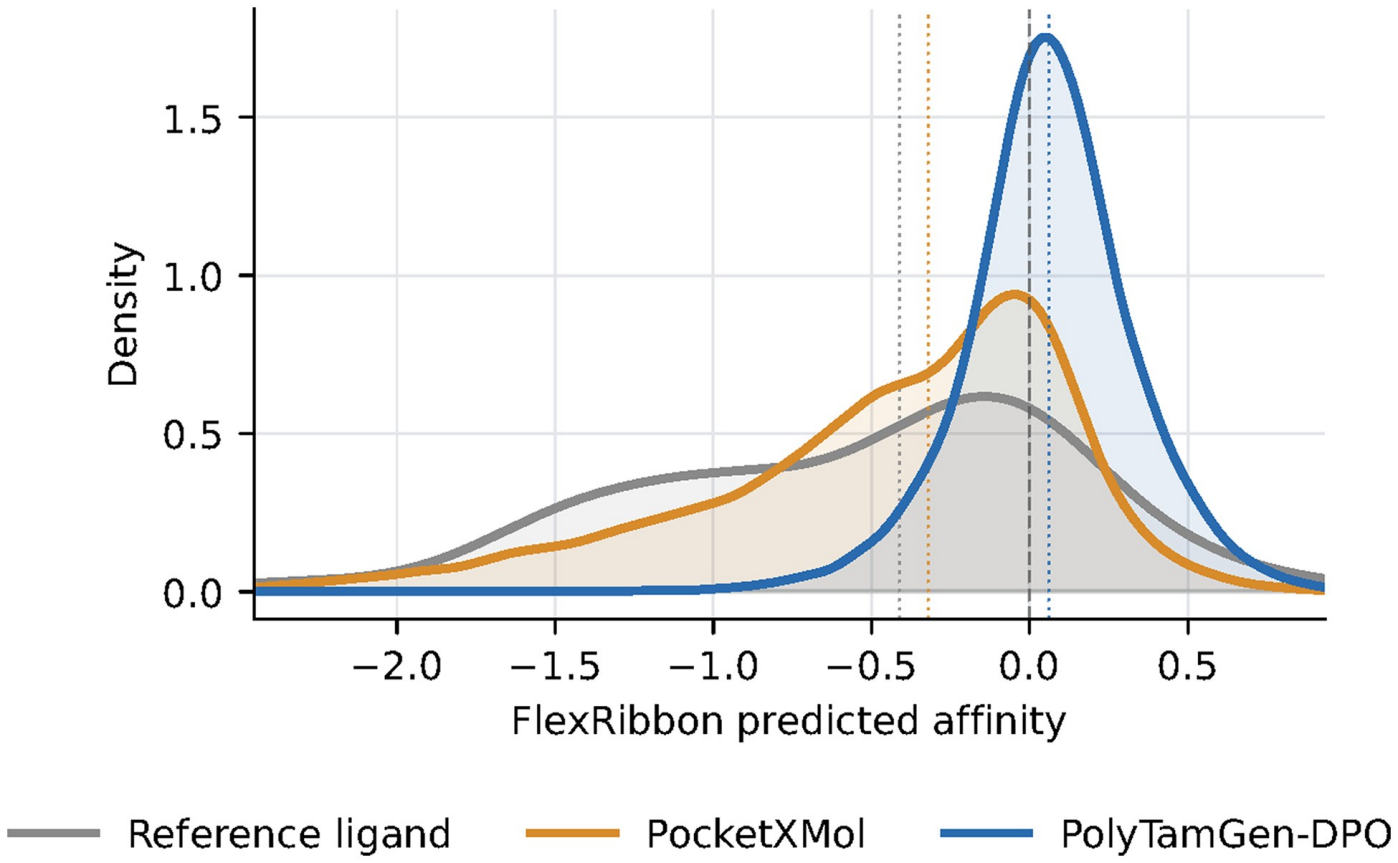
Average affinity-prediction density evaluation on T2 using FlexRibbon. Density distribution of aligned per-molecule average predictions across the two intended targets for reference ligands, PocketXMol candidates and PolyTamGen-DPO candidates. Curves shifted to the right indicate stronger predicted binding; dotted vertical lines mark method medians and the dashed vertical line marks zero predicted affinity. Larger values indicate stronger predicted binding in −log(μM) units.

**Table 2:** Overall results on the T3 benchmark (generating ligands that bind to three target proteins simultaneously).^a^

Method	AvgDock	SR_strict@-8.18	Validity	Diversity	QED	SA
TamGen	−6.737 ± 0.734	0.057 ± 0.069	0.998 ± 0.003	**0.866** ± **0.004**	0.488 ± 0.028	0.715 ± 0.008
PocketXMol	−6.788 ± 1.114	0.146 ± 0.165	**1.000** ± **0.001**	0.858 ± 0.053	0.531 ± 0.068	0.778 ± 0.054
PolyTamGen-SFT	−7.540 ± 0.793	0.239 ± 0.202	0.948 ± 0.053	0.865 ± 0.020	0.395 ± 0.075	0.742 ± 0.049
PolyTamGen-DPO	**−7.918** ± **0.819**	**0.405** ± **0.316**	0.966 ± 0.079	0.841 ± 0.019	**0.567** ± **0.068**	**0.806** ± **0.029**

a Entries report mean ± SD across target triplets. Bold values indicate the best mean performance for each metric: lower AvgDock and higher values for all other metrics.

As shown in Fig. 2, the average-affinity density distribution is shifted toward stronger predicted affinity for PolyTamGen-DPO. The mean predicted affinities of PolyTamGen-DPO, PocketXMol, and the reference ligands are 0.0594, −0.4458, and −0.5409, respectively. This trend is consistent with the docking-based evaluation and suggests that the observed gains are not specific to the docking metric alone.

The results on the T3 benchmark are summarized in Table 2. PolyTamGen-DPO reaches an ${\text{SR}}_{\text{strict}}$ of 40.5%, compared with 23.9% for PolyTamGen-SFT, 14.6% for PocketXMol, and 5.7% for TamGen. At the same time, the higher T3 headline score should not be interpreted as evidence that triple-target generation is easier than dual-target generation. T2 and T3 are different benchmark populations rather than matched difficulty-controlled extensions of one another: T2 contains 151 groups, 288 unique pockets, and 113 reference ligands, whereas T3 contains 59 groups, 97 unique pockets, and 23 reference ligands, making T3 smaller and more compositionally concentrated. In addition, triple-target groups are retained only when the same reference ligand is associated with all three target contexts, which may make T3 a more compositionally compatible subset than T2. A reference-ligand redocking analysis is also more favorable on T3 than on T2, both in average active-target docking over groups (−5.958 versus –5.582) and in hardest-target docking (−5.366 versus –5.161). Taken together, these statistics suggest that T3 is a smaller and more selectively retained benchmark population, which may help explain why its aggregate score is numerically higher despite the additional target constraint. Additional paired context-level comparisons on T2 are provided in Table 7, available as supplementary data at *Bioinformatics* online.

We further perform three complementary analyses on the T2 benchmark to examine different aspects of multitarget generation. First, a weakest-target evaluation tests whether the improvement in average docking scores is driven by one particularly favorable target; PolyTamGen remains the strongest method when performance is determined by the worse of the two target-specific docking scores (see Table 5, available as supplementary data at *Bioinformatics* online). Second, a cross-target conditioning stress test examines whether the model responds to the specified target pair by comparing molecules generated for the intended targets with those generated under an unrelated target condition. The intended conditioning consistently leads to better docking on the corresponding targets, supporting target-conditioned generation (see “Cross-target conditioning stress test (preliminary diagnostic)” in the Supplementary Information, available as supplementary data at *Bioinformatics* online). Third, we assess the conformational plausibility of the predicted docking poses and find that most molecules exhibit relatively small pose-energy gaps after local optimization, although substantial conformational strain remains in some cases (see “Conformational analysis” in the Supplementary Information, available as supplementary data at *Bioinformatics* online).

### 3.3 Structural case study

To further examine the structural adaptability of PolyTamGen, we analyze docking poses against the target pair LdtMt2 (PDB: 6IYW) and PBP5 (PDB: 6MKF). We selected this pair from the T2 benchmark because both proteins have resolved pocket structures and expose a clear geometric contrast: LdtMt2 contains a tunnel-shaped active site, whereas PBP5 contains a more open cleft-shaped site. This contrast makes the pair a useful qualitative test of whether one generated molecule can remain compatible with two distinct pocket shapes rather than only optimizing one target. For each method, we visualize a selected illustrative candidate for this target pair under the same docking/scoring protocol; the comparison is intended as a mechanistic illustration rather than experimental validation.

PolyTamGen (Fig. 3a) generates a candidate with strong joint docking scores, reaching −9.47 kcal/mol on 6IYW (Zhao *et al.* 2019) and −8.76 kcal/mol on 6MKF (Moon *et al.* 2018). In the 6IYW tunnel, the molecule adopts an extended, linear conformation to increase burial depth. In contrast, for the 6MKF cleft, the same scaffold uses flexible linkers to undergo a hinge-bending motion, wrapping around the pocket floor. Within this docking analysis, the two predicted poses illustrate that the same generated scaffold can adopt distinct conformations in the two pocket geometries.

**Figure 3:**
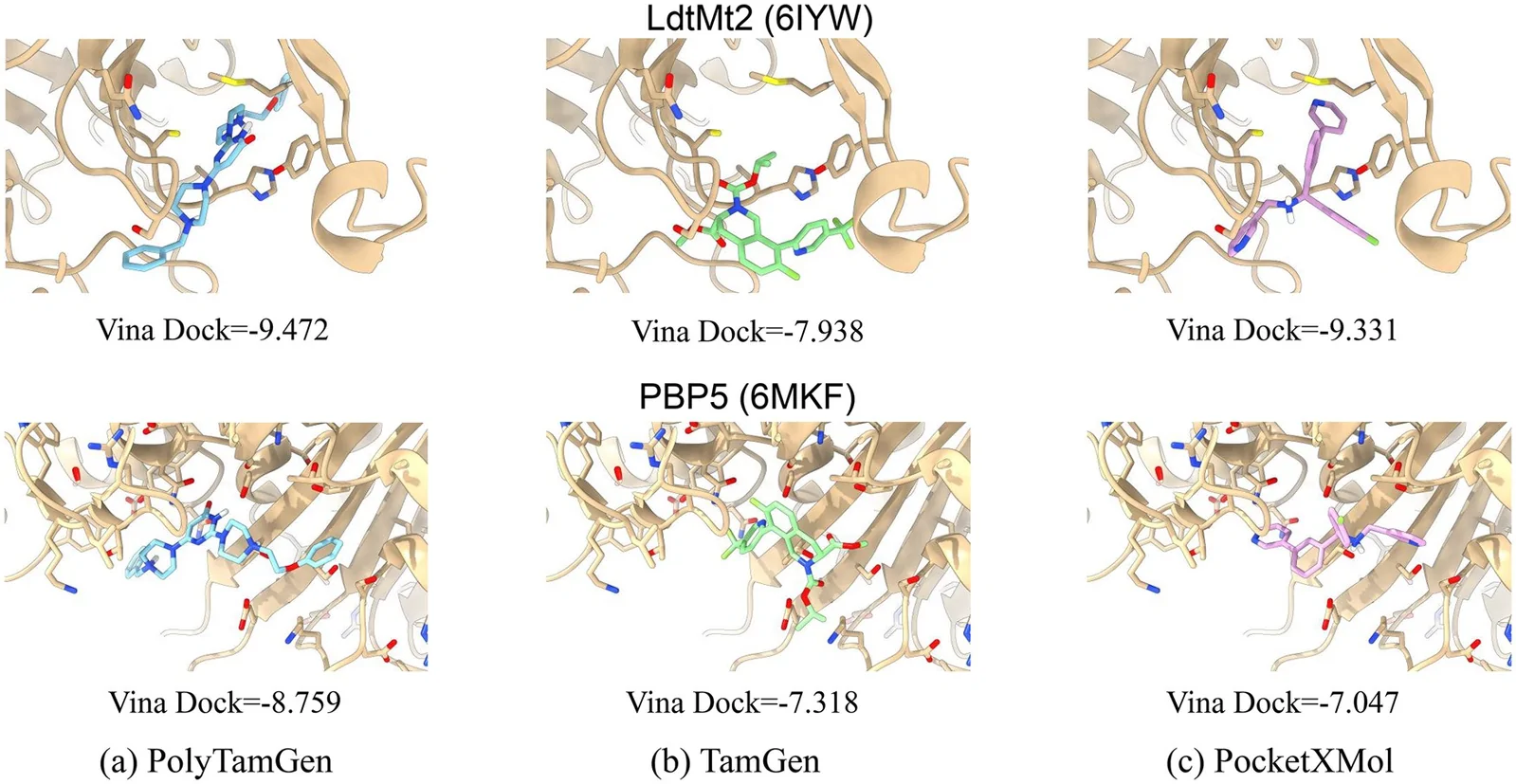
Structural comparison of generated dual-target candidates against the structurally distinct target pair LdtMt2 (6IYW) and PBP5 (6MKF). Rows correspond to the tunnel-shaped active site of LdtMt2 (top) and the cleft-shaped active site of PBP5 (bottom). (a) PolyTamGen: the selected candidate adopts distinct docked conformations in the two pockets and achieves favorable joint docking scores (−9.47/−8.76 kcal/mol). (b) TamGen: the selected candidate yields weaker scores in this example (−7.94/−7.32 kcal/mol). (c) PocketXMol: the selected candidate performs favorably in the 6IYW tunnel (−9.33 kcal/mol) but less favorably in the 6MKF cleft (−7.05 kcal/mol), with the predicted pose indicating poorer geometric compatibility with the latter pocket.

The TamGen candidate (Fig. 3b) yields balanced but weaker scores (−7.94 and −7.32 kcal/mol). The visualization suggests that its bulky, rigid scaffold occupies the general pocket volume but does not access the same deep subsites as the illustrated PolyTamGen candidate. In this example, the PocketXMol candidate (Fig. 3c) performs well on 6IYW (−9.33 kcal/mol) but less favorably on 6MKF (−7.05 kcal/mol); the predicted pose indicates poorer geometric compatibility with the latter pocket. This qualitative comparison illustrates distinct predicted pose behavior for the selected candidates under the current docking protocol, without implying a general failure mode of single-target generation methods.

## 4 Conclusions and discussion

In this paper, we introduced PolyTamGen, a sequence-based framework for joint multitarget molecular generation that reformulates polypharmacological design as conditioned sequence modeling. To address the scarcity of true multitarget structural data, we curated a comprehensive ligand-centric dataset comprising 4.9 million bioactivity records and 1.6 million unique ligands from ChEMBL and BindingDB. Architecturally, PolyTamGen integrates an ESM3-based protein encoder, an adaptor, and a set-wise interaction module with NatureLM, and is refined through SFT followed by direct preference optimization.

Experimental results on the proposed docking-based benchmarks show that PolyTamGen achieves stronger joint compatibility metrics than the compared baselines in both the T2 setting, where generated molecules are required to bind two target proteins, and the T3 setting, where they are required to bind three target proteins. These results suggest that explicit target-set conditioning and cross-target interaction modeling provide a useful computational strategy for multitarget molecule generation.

We are aware that the current method has several limitations to be addressed. PolyTamGen should be understood as an early-stage computational framework for hypothesis generation rather than a complete solution to real-world medicinal-chemistry optimization. The present model conditions only on desired active targets and has no antitarget role, so it cannot impose a controllable “bind target A while avoiding target B” objective. This study focuses on satisfying multitarget binding constraints and does not establish experimentally validated polypharmacology. In addition, the training and evaluation signals are not perfectly aligned, as the model is trained from aggregated bioactivity labels whereas the main benchmark relies on docking-based proxy metrics. Finally, the ligand-centric dataset is constructed from heterogeneous assay sources and may therefore contain residual noise despite careful filtering.

Important future directions include explicit antitarget and selectivity-aware objectives, incorporation of richer assay context, and downstream experimental validation of the generated candidates. These extensions would further strengthen the practical utility of sequence-based multitarget molecular generation for early-stage drug discovery.

## Supplementary Material

btag766_Supplementary_Data

## Data Availability

The code is available at https://github.com/bjzgcai/PolyTamGen. The processed data, model checkpoints, and benchmark results are available at Zenodo: https://doi.org/10.5281/zenodo.20594128.
