# Supplementary material for: Polypharmacological target-aware molecule generation
Source: Bioinformatics. 2026 Oct 12;42(10):btag766. doi: 10.1093/bioinformatics/btag766 (PMC13715970; doi:10.1093/bioinformatics/btag766)
Supplement: Supplementary file 1 [file btag766_supplementary_data.pdf]

# Polypharmacological Target-Aware Molecule Generation Supplementary Information

Kehan Wu[1,2,†], Wei Zhang[1,2,†], Han Guo[3], Renhe Liu[3], Yu Shi[2], Peiran Jin[2]
Haiguang Liu[2], Enhong Chen[1], Tao Qin[2], Zequn Liu[2,*] and Yingce Xia[2,*]

[1]University of Science and Technology of China, Hefei, Anhui, China
[2]Zhongguancun Academy, Beijing 100094, China
[3]Global Health Drug Discovery Institute, Beijing, China
[*]Corresponding authors: liuzequn@bza.edu.cn; yingce.xia@gmail.com
[†]These authors contributed equally to this work.

Supplementary Tables S1–S7
Supplementary Figures S1–S3
Supplementary Notes

## Supplementary Tables

| Method | $SR_{strict}$@−8.18 | $SR_{strict}$@−7.0 |
|---|---|---|
| **T2** | | |
| TamGen | 0.073 | 0.306 |
| PocketXMol | 0.163 | 0.356 |
| PolyTamGen-Single | 0.148 | 0.380 |
| PolyTamGen-SFT | 0.226 | 0.475 |
| PolyTamGen-DPO | 0.331 | 0.648 |
| **T3** | | |
| TamGen | 0.057 | 0.348 |
| PocketXMol | 0.146 | 0.413 |
| PolyTamGen-SFT | 0.239 | 0.546 |
| PolyTamGen-DPO | 0.405 | 0.767 |

**Table S1:** Strict-success rates under the two docking-score cutoffs. Following DecompDiff, QED > 0.25 and normalized SA > 0.59 are held fixed in both columns. The DecompDiff Vina cutoff of −8.18 kcal/mol is the primary endpoint used in the main tables, while −7.0 kcal/mol is retained as a more permissive threshold-sensitivity comparison.

| Benchmark | Method | Random-10 | Top-10 | Random-20 | Top-20 |
|---|---|---|---|---|---|
| T2 | TamGen | 7.2 | 37.7 | 7.2 | 32.3 |
| | PocketXMol | 16.3 | 42.7 | 16.3 | 40.5 |
| | PolyTamGen-Single | 14.9 | 42.7 | 14.8 | 40.9 |
| | PolyTamGen-SFT | 22.6 | 44.5 | 22.7 | 42.4 |
| | PolyTamGen-DPO | **32.9** | **58.1** | **33.0** | **53.9** |
| T3 | TamGen | 5.6 | 44.4 | 5.7 | 35.4 |
| | PocketXMol | 14.8 | 51.9 | 14.6 | 48.2 |
| | PolyTamGen-SFT | 23.9 | 47.8 | 23.8 | 45.5 |
| | PolyTamGen-DPO | **40.5** | **71.7** | **40.5** | **67.4** |

**Table S2:** Candidate-budget sensitivity of the strict success rate (%). All values use the primary Vina cutoff of −8.18 kcal/mol, QED > 0.25, and normalized SA > 0.59. Top-$N$ denotes docking-based reranking by the weakest intended-target score, with AvgDock and candidate rank used to break ties. Random-$N$ denotes sampling without replacement, averaged over 100 repeats. Both protocols select from candidates with finite AvgDock values, consistent with the denominator of $SR_{strict}$, before target-set-equal aggregation. PolyTamGen-DPO remains highest at both reported budgets. All 151 T2 target sets were evaluable. For T3, 59 target sets were evaluable for each method except PocketXMol, for which 57 contained at least one candidate with finite AvgDock.

| Reward setting | AvgDock | Validity | Diversity | QED | Normalized SA |
|---|---|---|---|---|---|
| $\lambda = 1$ | -7.653 | 0.980 | 0.843 | 0.538 | 0.808 |
| $\lambda = 5$ (main model) | -7.642 | 0.968 | 0.837 | 0.565 | 0.805 |
| $\lambda = 10$ | -7.526 | 0.974 | 0.831 | 0.660 | 0.822 |

**Table S3:** Reward-coefficient sensitivity on T2. The reported PolyTamGen-DPO model uses $\lambda = 5$; $\lambda = 1$ and $\lambda = 10$ are comparison settings. Synthetic accessibility is not an explicit reward term.

| Method | AvgDock | $SR_{strict}$@-7.0 | Validity | Diversity | QED | SA |
|---|---|---|---|---|---|---|
| PolyTamGen-SFT | -7.347 | 0.475 | 0.959 | 0.864 | 0.408 | 0.753 |
| `SetWiseOff` | -7.327 | 0.387 | 0.954 | 0.867 | 0.394 | 0.763 |
| `LinearAdaptor` | -6.794 | 0.348 | 0.959 | 0.865 | 0.469 | 0.772 |

**Table S4:** Module-level ablations on T2, reported at the −7.0 kcal/mol sensitivity cutoff. We compare PolyTamGen-SFT with two controls to assess the contribution of the explicit set-wise interaction module and the richer target adaptor. `SetWiseOff` keeps the ESM3 encoder and the original Q-Former-style protein adaptor, but disables cross-target self-attention so that target tokens no longer exchange information. `LinearAdaptor` replaces the Q-Former-style adaptor with a simple linear projection, removing learned query-based compression and cross-attention. Both controls reduce the binding-oriented metrics. The primary −8.18 endpoint for the complete model and baselines is given in Table S1; the ablation values here are retained as a complementary sensitivity analysis.

| Method | WorstDock | $SR_{strict,worst}$@−8.18 | $SR_{strict,worst}$@−7.0 |
|---|---|---|---|
| TamGen | -6.017 | 0.033 | 0.176 |
| PocketXMol | -5.836 | 0.074 | 0.211 |
| PolyTamGen-Single | -6.421 | 0.064 | 0.206 |
| PolyTamGen-SFT | -6.753 | 0.108 | 0.287 |
| PolyTamGen-DPO | **-7.048** | **0.186** | **0.454** |

**Table S5:** Strict weakest-target evaluation on T2. To avoid average docking scores hiding failure on one desired target, each molecule is evaluated by its weakest desired-target docking outcome rather than by the average across desired targets. We report the primary −8.18 kcal/mol cutoff and the −7.0 kcal/mol sensitivity cutoff, using QED > 0.25 and normalized SA > 0.59 in both cases. Under this balance-aware criterion, PolyTamGen-DPO remains the strongest method at both cutoffs, indicating that its gains are not merely an averaging artifact.

| | **PolyTamGen-Struct** | **PolyTamGen-SFT** |
|---|---|---|
| Data Records | 21,040 | $\sim$4.9 million |
| Unique Ligands | 2,047 | $\sim$1.65 million |
| AvgDock | -5.610 $\pm$ 1.280 | -7.347 $\pm$ 1.017 |
| $SR_{strict}$@-7.0 | 0.148 $\pm$ 0.210 | 0.475 $\pm$ 0.290 |

**Table S6:** Impact of data regime on multi-target generation, with strict-success rates reported at the $-7.0$ kcal/mol sensitivity cutoff. We compare PolyTamGen-Struct, a structure-only variant trained on the curated PDB-derived dataset $\mathcal{D}_{\mathrm{PDB}}$, with PolyTamGen-SFT, which is trained on the large-scale bioactivity dataset $\mathcal{D}_{\mathrm{bio}}$. Although $\mathcal{D}_{\mathrm{PDB}}$ provides direct structural supervision, it is data-limited in this setting, containing 21,040 ligand–target records and 2,047 unique ligands, whereas $\mathcal{D}_{\mathrm{bio}}$ contains approximately 4.9 million records and 1.65 million ligand profiles. The primary endpoint is reported in Table S1; these data-regime values are retained to show the sensitivity of the comparison to the original cutoff.

| PolyTamGen-DPO vs. | BothTargetsBetterRate | AvgBetterRate |
|---|---|---|
| PolyTamGen-Single | 0.974 | 0.987 |
| TamGen | 0.987 | 0.993 |
| PocketXMol | 0.868 | 0.907 |

**Table S7:** Paired context-level comparisons on T2 using the canonical molecule-level scored records. For each method and target-pair context, means are computed over molecules with finite docking scores for both intended targets. BothTargetsBetterRate is the fraction of contexts in which PolyTamGen-DPO has lower group-level mean docking scores on both intended targets than the comparator; AvgBetterRate is the fraction in which the corresponding two-target average is lower. Both metrics are threshold-independent.

## Supplementary Figures

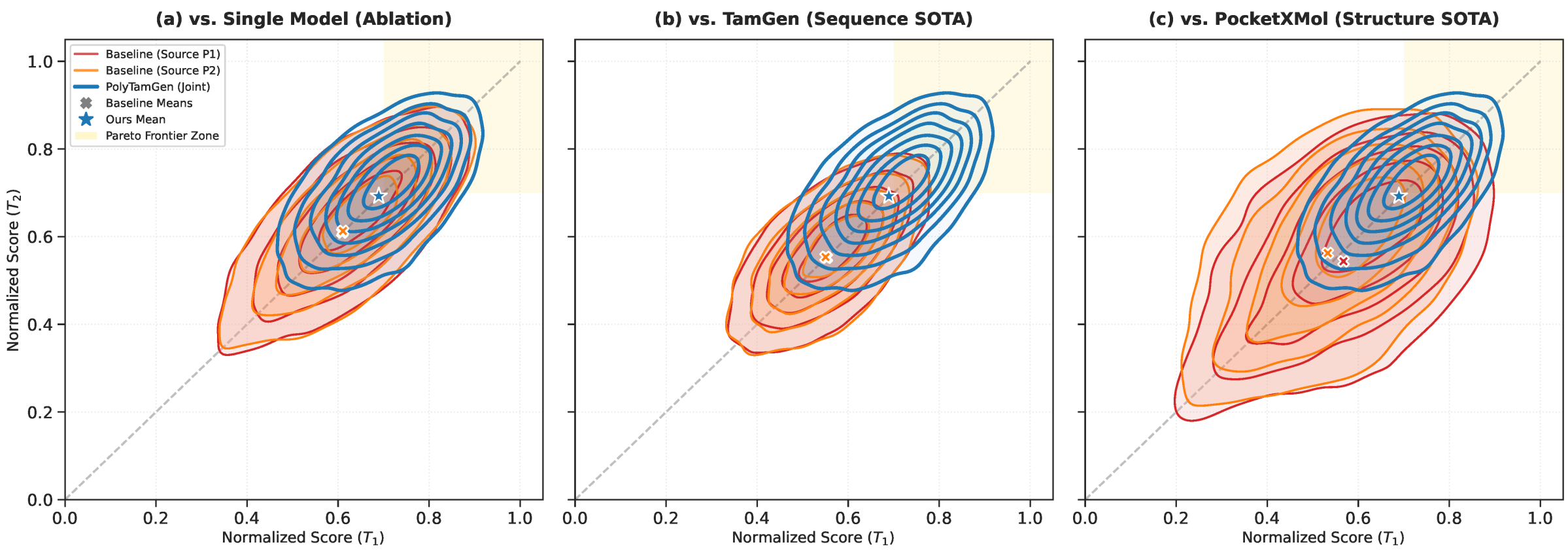


**Figure S1: Source-separated joint binding affinity landscapes.** The 2D kernel density plots visualize the normalized docking scores across all test pairs. Baselines are decomposed into sub-populations generated for Target 1 (Red, Source P1) and Target 2 (Orange, Source P2), with their respective centroids marked by 'X'. The source-separated views reveal target-specific shifts that are obscured by pooling the two baseline populations. PolyTamGen (Blue) generates a unified distribution whose centroid (Blue Star) and density mass are shifted toward the high-affinity joint region (gold background), consistent with stronger simultaneous docking under the normalized view.

*Alt text: Four density plots compare normalized dual-target docking distributions for the baselines and PolyTamGen. The PolyTamGen distribution is shifted toward the high-affinity joint region.*

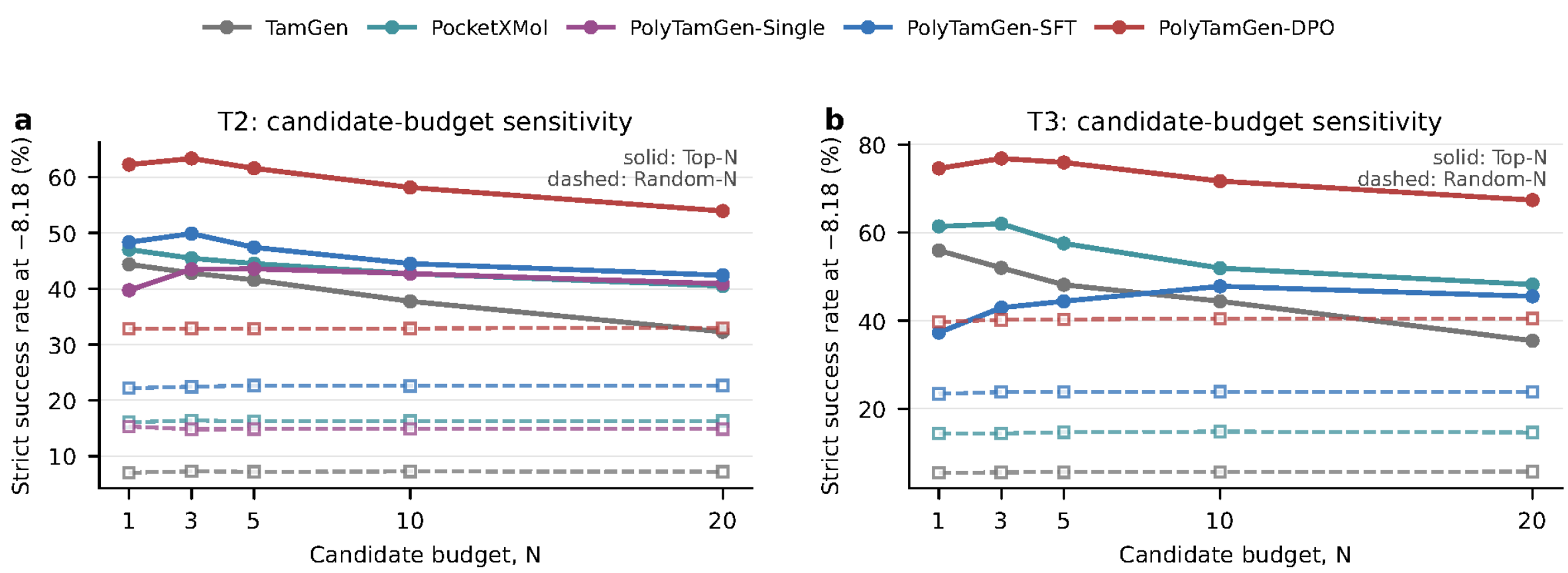


**Figure S2: Candidate-budget sensitivity.** Strict success rates on (a) T2 and (b) T3 for candidate budgets $N = 1, 3, 5, 10, 20$. Solid lines show docking-reranked Top-$N$ candidates, whereas dashed lines show Random-$N$ candidates sampled without replacement and averaged over 100 repeats. Candidate selection is restricted to molecules with finite AvgDock values, consistent with the metric denominator. PolyTamGen-DPO remains highest across all evaluated candidate budgets.

*Alt text: Two line plots show candidate-budget sensitivity on the T2 and T3 benchmarks. Solid lines show docking-reranked Top-N results and dashed lines show Random-N results. PolyTamGen-DPO has the highest strict success rate at every displayed candidate budget.*

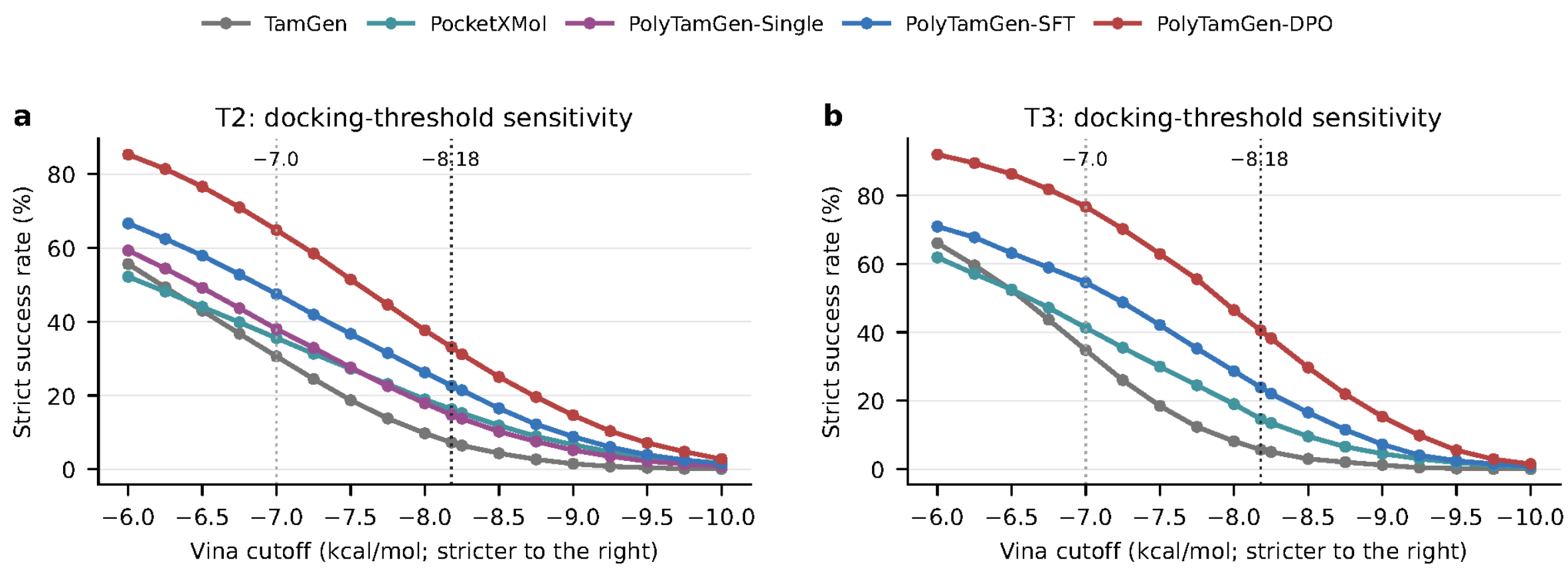


**Figure S3: Docking-threshold sensitivity.** Strict success rates on (a) T2 and (b) T3 for the complete evaluated candidate pools as the Vina cutoff varies from −6.0 to −10.0 kcal/mol, with the QED and normalized-SA criteria held fixed. Vertical lines mark −7.0 and the primary DecompDiff cutoff of −8.18 kcal/mol. PolyTamGen-DPO remains highest throughout the tested cutoff range, although the relative ordering among the other methods varies.

*Alt text: Two line plots show docking-threshold sensitivity on the T2 and T3 benchmarks. PolyTamGen-DPO has the highest strict success rate throughout the tested docking-cutoff range.*

## Supplementary Notes

**Analysis of joint distribution shift.** To investigate the mechanism behind the performance gains, we visualize the joint binding affinity landscape on the T2 benchmark. Figure S1 presents the 2D distribution of docking scores, where the x-axis and y-axis represent the normalized affinities for the first ($P_1$) and second ($P_2$) targets, respectively. To ensure comparability across heterogeneous target pairs, raw scores are mapped to a $[0, 1]$ scale via per-pair min-max normalization.

**Deconstructing baseline balance.** The pooled distributions of the single-target baselines appear comparatively balanced around the diagonal. When the same outputs are separated by source target, however, the Target 1-generated population is shifted toward stronger $T_1$ affinity and the Target 2-generated population toward stronger $T_2$ affinity. This source dependence shows that pooling can mask target-specific generation bias in the single-target baselines.

**Joint optimization trend.** In contrast, PolyTamGen generates a unified population that follows the bisector more closely and is shifted toward the high-affinity joint region under the normalized docking view. The density analysis is descriptive. Its shift toward the high-affinity joint region is consistent with the aggregate and weakest-target results.

**Weakest-target diagnostics.** Average docking can hide a weak interaction with one desired target, so we also evaluate each T2 molecule by its worst desired-target docking score. Table S5 shows that PolyTamGen-DPO remains strongest under this criterion. The AvgDock improvement is therefore not driven only by one strong target.

**Cross-target conditioning stress test (preliminary diagnostic).** To examine whether molecular generation responds to the intended target condition, we performed a cross-target conditioning stress test on T2. For each desired pair $(A, B)$, we selected a distinct, non-intended target $C$ from the available single-target contexts. Molecules generated under $(A, B)$ conditioning and under $C$ conditioning were evaluated on the same $A$ and $B$ pockets. Candidates were paired by rank within each context, without using docking results for candidate selection.

Across 151 contexts and 1,300 matched-rank comparisons, the context-averaged mean docking score on $A$ and $B$ was $-7.670$ kcal/mol for $(A, B)$-conditioned molecules and $-6.969$ kcal/mol for $C$-conditioned molecules. The mean conditioning advantage was 0.701 kcal/mol (paired-context bootstrap 95% CI, 0.613–0.790), and the $(A, B)$-conditioned molecules performed better in 92.7% of contexts.

These results support a target-conditioning effect under the current computational protocol. The model uses active-target supervision without explicit anti-target conditioning, and selectivity-aware generation remains future work.

**Pocket definition and structural split.** Pockets are defined by including protein residues for which any atom lies within 10.0 Å of any atom of the selected co-crystallized ligand; all atoms of each selected residue are retained. Water, ions, the bound ligand, and other non-protein hetero residues are excluded. Structural records are grouped by the unordered pair of exact pocket identifiers, assigning each pocket-pair context to one subset; this exact pair grouping defines T2. T3 is constructed separately by aggregating pocket contexts associated with the same reference ligand in the structural test records and forming three-pocket contexts for ligands represented across at least three such pockets. No geometric- or sequence-similarity clustering is used to define the T2 split groups. Reference ligands may recur in different structural contexts, so the benchmarks are interpreted at the level of their respective target-context combinations. The reference ligand associated with each structural record identifies the target context; it is not used as a chosen or rejected DPO response, because both preference responses are generated by the reference policy.

**Protein representation.** We mapped protein chains from the available pocket files to canonical UniProt accessions to describe the protein content of $\mathcal{D}_{\mathrm{PDB}}$. Of the 21,040 structural records, 20,981 had available pocket files containing protein residues and were eligible for this chain-level analysis. The resulting mapping identified 7,389 distinct canonical UniProt accessions. This count is separate from the 9,607 entry-level UniProt cross-references. Some eligible pocket files still lack a chain-level UniProt mapping. These are mapping-specific counts and do not constitute a complete census of biological proteins.

**Structural split and pocket-position sequence conservation.** The ligand-scaffold split used for $\mathcal{D}_{\mathrm{bio}}$ does not enforce protein independence. We therefore analyzed sequence relatedness between the structure-defined T2 pockets and active-protein references in the released supervised training and validation profiles. Using residue-level SIFTS mappings (Dana et al., 2019), we linked 274 of the 288 T2 pockets to full UniProt sequences; pockets without a reliable mapping were excluded.

Full UniProt sequences were searched with MMseqs2 against the supervised reference proteins using sensitivity 7.5, an E-value cutoff of $10^{-5}$, and a minimum alignment length of 20 residues. The reference sets contained 3,419 training-active and 1,373 validation-active accessions, including 1,371 shared accessions and 3,421 accessions in the deduplicated union. Identity refers to the reported local alignment, and query and target coverage were recorded separately. No retained sequence hit was found for 119 of the 274 analyzable pockets (43.4%); these queries were excluded from identity denominators.

For each retained hit, we projected the mapped test-pocket positions through the gapped alignment and calculated pocket coverage, identity among aligned pocket residues, and identity over all selected-chain pocket residues. Among the 141 pockets with at least one alignment covering 80% or more of the defined pocket positions, no retained pair combined local-alignment identity below 0.3, pocket coverage of at least 0.8, and aligned-pocket identity of at least 0.8. Exact-accession and homologous relationships were present among the retained hits. T2 is therefore reported as a held-out exact pocket-pair-context benchmark and is not protein-disjoint. Because the supervised reference proteins lack uniformly defined structural pockets, this analysis measures sequence conservation at the T2 pocket positions. It does not measure direct three-dimensional pocket similarity.

**Ligand overlap and generated-molecule comparison.** Because the structural dataset is split by pocket-pair combinations rather than ligand identity, reference ligands can occur in more than one structural subset. We therefore explicitly quantified ligand-level overlap between the training and evaluation sets.

For the T2 benchmark, 110 of the 113 unique reference ligands also occur in the structural training set. This high overlap is a consequence of the construction of $D_{\mathrm{PDB}}$: the dataset is built from ligands observed across multiple target structures, while the held-out unit is the pocket-pair combination rather than the ligand itself. Thus, T2 should be interpreted as evaluating generalization to held-out target/pocket combinations, not to unseen ligands.

Although reference ligands can overlap across the structural subsets, we found little evidence that the model directly reproduces structural-training ligands. Exact matches occurred in only 3 of 14,533 valid unique T2 outputs and none of the 5,685 T3 outputs, while the median maximum Morgan similarities to the structural-training ligands were 0.296 and 0.293, respectively. Thus, reference-ligand overlap in the benchmark does not translate into substantial direct copying in the generated outputs.

Public structure-based benchmarks make a similar distinction between the split axis and ligand novelty. CrossDocked2020 grouped pockets into three folds using ProBiS pocket similarity, and its authors noted that ligand similarity could remain across folds (Francoeur et al., 2020). The downstream CrossDocked 100k/100 generation benchmark instead used 30%

protein-sequence clustering and was subsequently reused by Pocket2Mol (Luo et al., 2021; Peng et al., 2022). A version-locked audit found exact ligand overlap in at least 39 of 100 test complexes. It also found scaffold overlap in at least 53 of 87 test complexes with a non-empty Murcko scaffold. Exact identity used RDKit canonical isomeric SMILES derived from RCSB Chemical Component Dictionary records, and scaffolds were computed from the same molecules. Fifteen unusual training components could not be canonicalized, so both counts are lower bounds. These results illustrate that protein- or pocket-based structural splits can retain ligand overlap and support reporting ligand novelty as a separate benchmark property.

**Threshold sensitivity.** Figure S3a,b evaluates the complete candidate pools over Vina cutoffs from −6.0 to −10.0 kcal/mol, with the candidate sets, QED criterion, normalized-SA criterion, denominator, and target-set aggregation held fixed. Table S1 reports the exact values at −8.18 and −7.0 kcal/mol. The −8.18 kcal/mol cutoff follows the DecompDiff benchmark convention and was not calibrated specifically for this multi-target setting. As expected, the more permissive −7.0 cutoff increases the absolute success rates. PolyTamGen-DPO remains highest throughout the tested range, while the ordering among the other methods changes at several cutoffs. Supplementary ablation and data-regime tables using −7.0 are retained as sensitivity analyses.

**Candidate-budget sensitivity.** The main benchmark evaluates all available candidates in each complete pooled output against every intended target. We also compared docking-reranked Top-$N$ selection with Random-$N$ sampling at $N = 1, 3, 5, 10, 20$ (Figure S2a,b). Random-$N$ sampling was performed without replacement and repeated 100 times within each target set. Both protocols used candidates with finite AvgDock, consistent with the denominator of $\mathrm{SR}_{\mathrm{strict}}$, and target-set results were averaged with equal weight. Table S2 reports the Top-10 and Top-20 values. PolyTamGen-DPO remains highest for both protocols at every evaluated budget. The evaluate-all results remain the primary comparison; Top-$N$ quantifies the effect of docking-based reranking under a fixed candidate budget.

**Conformational analysis.** We additionally report a conformational strain / pose-energy-gap diagnostic using Open Babel/MMFF94 on the same 151 target pairs with 5 molecules per pair. The reported internal energies are MMFF94 energies in kcal/mol. The mean pose-energy gap is 2.377 kcal/mol, the mean worst strain is 113.143 kcal/mol, the fraction with pose gap $\leq 5$ kcal/mol is 0.918, and the fraction with worst strain $\leq 10$ kcal/mol is 0.194. Small differences between the two predicted pose energies are common, but the high absolute worst-strain values and low fraction below 10 kcal/mol show that this diagnostic does not establish physical consistency. We therefore report it as a limitation-aware energetic diagnostic rather than validation of the docking poses.

**Architectural motivation.** To clarify the role of the explicit set-wise interaction module and the richer target adaptor, we compare PolyTamGen-SFT against two module-level controls on T2. `SetWiseOff` keeps the ESM3 encoder and the original Q-Former-style protein adaptor, but disables the Set-Wise Interaction module so that target tokens no longer exchange information through cross-target self-attention. `LinearAdaptor` replaces the Q-Former-style adaptor with a simple linear projection-based adaptor, removing the learned query-based compression and cross-attention mechanism. Table S4 shows that both controls degrade the two reported binding-oriented metrics under this ablation setting. The `SetWiseOff` control remains close to PolyTamGen-SFT in mean AvgDock but is lower in $\mathrm{SR}_{\mathrm{strict}}$, suggesting that explicit cross-target interaction mainly improves the rate at which candidates satisfy the joint strict-success criterion. These results suggest that explicit cross-target interaction and richer target conditioning both contribute meaningfully to dual-target generation performance.

**DPO reward sensitivity.** We further examine the effect of the QED coefficient $\lambda$ in the DPO reward (Table S3). The reported PolyTamGen-DPO model uses $\lambda = 5$, while $\lambda = 1$ and $\lambda = 10$ are included as lower- and higher-weight comparison settings. Across the tested settings, AvgDock, validity, and diversity remain broadly comparable, while QED varies with the relative weight assigned to drug-likeness. Normalized SA is reported as an evaluation metric but is not explicitly included in the reward. Across the pooled molecule-level scored records with finite QED and normalized SA values, QED and normalized SA show moderate associations (T2 Pearson $r = 0.428$, Spearman $\rho = 0.414$; T3 Pearson $r = 0.416$, Spearman $\rho = 0.411$). We report this SA variation as an empirical association. SA is not included in the reward and is not guaranteed to improve when the QED weight changes.

**Data scale versus structure-only supervision.** As introduced in the main text, we also curated a multi-target dataset from the PDB (denoted as $\mathcal{D}_{\text{PDB}}$). However, this regime is severely data-limited, containing only 2,047 unique ligands and 21,040 ligand-target records. To benchmark the role of data regime, we implemented a structure-only variant trained from $\mathcal{D}_{\text{PDB}}$, denoted as PolyTamGen-Struct, while PolyTamGen-SFT is trained from the large-scale bioactivity regime $\mathcal{D}_{\text{bio}}$. As shown in Table S6, PolyTamGen-Struct achieves a Strict Success Rate of only 14.8% and an AvgDock of $-5.61$, compared with 47.5% and $-7.34$ for PolyTamGen-SFT. This comparison is consistent with a substantial data-scale disadvantage for the structure-only regime in the present multi-target setting.

**Additional paired comparisons.** To complement the aggregate benchmark metrics, we further report paired context-level comparisons on T2. For each method and target-pair context, group-level means are computed over molecules with finite docking scores for both intended targets. BothTargetsBetterRate measures the fraction of contexts for which PolyTamGen-DPO achieves lower mean docking scores on both intended targets than the comparator, while AvgBetterRate measures the fraction for which the corresponding two-target average is lower. These threshold-independent diagnostics show consistently favorable paired comparisons against the single-target baselines (Table S7).

**Implementation details.** We adopted the 1B-parameter NatureLM re-implementation as the molecule generator. For protein feature extraction, we employed ESM3-C (600M) with frozen weights. To bridge the modality gap, we utilized a Q-Former adaptor comprising 2 decoder layers, which compresses the protein features using $S = 32$ learnable queries. To capture cross-target dependencies, the compressed tokens are processed by a Set Transformer consisting of 2 layers with 8 attention heads. RoPE positional indices are reset within each protein block so that the interaction module does not assign distinct absolute block-position indices to targets solely because of their concatenation order. For the sampled SFT target subset, we randomly sample 1, 2, or 3 target proteins with equal probability. To ensure training efficiency, we applied LoRA ($r = 8$, $\alpha = 32$) to the LLM backbone. The model was fine-tuned on 8 NVIDIA A100 (80GB) GPUs for 20 epochs with a global batch size of 128 and a learning rate of $1 \times 10^{-4}$ using a cosine schedule with 1,000 warmup steps. For DPO preference construction, approximately 100 SFT-generated raw candidates were considered per structural target context. Candidates without a valid molecule or complete docking scores for all intended targets were excluded before reward ranking; among the remaining candidates, the highest- and lowest-reward molecules were selected as the preferred and rejected responses, respectively. Subsequently, in the DPO phase, we optimized the model for 2 epochs using a batch size of 8, a learning rate of $5 \times 10^{-6}$, and $\beta = 0.1$. We denote the models obtained from the SFT and DPO stages as PolyTamGen-SFT and PolyTamGen-DPO, respectively. Unless otherwise specified, we recommend using PolyTamGen-DPO as the default model. During inference, we

employ nucleus sampling (top-$p = 0.95$) with a temperature of 1.0 to balance chemical diversity and adherence to pharmacophoric constraints.

**Methodological limitations.** This work has several limitations. First, the model is trained from aggregated bioactivity labels, whereas the main benchmark uses docking-based proxy metrics. Benchmark performance under this protocol does not replace experimental validation. Second, aggregation and binarization of heterogeneous assay sources may introduce residual noise. Third, training uses active-target supervision and does not explicitly optimize anti-target avoidance, toxicity or selectivity. The cross-target stress test measures target-conditioned behavior under the current protocol. It does not test controllable selectivity.

**Ethical considerations and practical use.** The proposed framework is intended as an early-stage hypothesis-generation tool, not as a standalone decision-maker for therapeutic development. Because generative models for bioactive molecules may be misused, any practical deployment requires expert human review, downstream safety screening, application-specific constraints, and experimental validation. Computational generation is one component of a broader, safety-aware discovery workflow and does not replace medicinal-chemistry judgment.
